# *PSMD1* and *PSMD2* regulate HepG2 cell proliferation and apoptosis via modulating cellular lipid droplet metabolism

**DOI:** 10.1186/s12867-019-0141-z

**Published:** 2019-11-08

**Authors:** Yanjie Tan, Yi Jin, Xiang Wu, Zhuqing Ren

**Affiliations:** 1 Key Laboratory of Swine Genetics and Breeding of Ministry of Agriculture & Key Laboratory of Agriculture Animal Genetics, Breeding and Reproduction of Ministry of Education, College of Animal Science, Huazhong Agricultural University, Wuhan, 430070 Hubei People’s Republic of China; 2 Bio-Medical Center of Huazhong Agricultural University, Wuhan, 430070 Hubei People’s Republic of China

**Keywords:** *PSMD1*, *PSMD2*, Lipid droplet, Cell proliferation

## Abstract

**Background:**

Obesity and nonalcoholic steatohepatitis (NASH) are well-known risk factors of hepatocellular carcinoma (HCC). The lipid-rich environment enhances the proliferation and metastasis abilities of tumor cells. Previous studies showed the effect of the ubiquitin–proteasome system (UPS) on tumor cell proliferation. However, the underlying mechanism of UPS in regulating the proliferation of lipid-rich tumor cells is not totally clear.

**Results:**

Here, we identify two proteasome 26S subunits, non-ATPase 1 and 2 (*PSMD1* and *PSMD2*), which regulate HepG2 cells proliferation via modulating cellular lipid metabolism. Briefly, the knockdown of *PSMD1* and/or *PSMD2* decreases the formation of cellular lipid droplets, the provider of the energy and membrane components for tumor cell proliferation. Mechanically, *PSMD1* and *PSMD2* regulate the expression of genes related to de novo lipid synthesis via p38-JNK and AKT signaling. Moreover, the high expression of *PSMD1* and *PSMD2* is significantly correlated with poor prognosis of HCC.

**Conclusion:**

We demonstrate that *PSMD1* and *PSMD2* promote the proliferation of HepG2 cells via facilitating cellular lipid droplet accumulation. This study provides a potential therapeutic strategy for the treatment of lipid-rich tumors.

## Background

Hepatocellular carcinoma (HCC) is one of the most frequent causes of cancer-related deaths worldwide [1]. The chronic liver damage and inflammation caused by chronic hepatitis B virus or hepatitis C virus infections are common reason for HCC development. However, the incidence of nonviral HCC is rapidly increasing, especially in developed countries. Non-alcoholic fatty liver disease (NAFLD) and non-alcoholic steatohepatitis (NASH), which are usually enhanced by obesity, are well-known risk factors of HCC [2, 3]. Patients with obesity and NAFLD/NASH show an increased intake of dietary fatty acids (FAs), and meanwhile, insulin resistance enhances lipolysis of adipose tissue, which causes an increased exogenous FA supply and results in the development of a “lipid-rich” environment for hepatocytes. The lipid-rich environment is considered to promote the proliferation and metastasis of tumor cells [4–6]. Compared to normal cells, the tumor cells usually uptake larger amount of lipids, accompanied by enhanced lipogenesis and cholesterol (CH) production and increased fatty acid (FA) β-oxidation [7, 8]. Furthermore, FA synthetases such as FASN and ACS play an important role in tumor cell proliferation and tumorigenicity [9, 10]. Lipids are required for the proliferation process. For example, the ovarian tumor cells can obtain lipids from the adipocytes grown in coculture experiments [11]. Besides enhanced lipid uptake, tumor cells often accumulate a larger amount of cellular lipid droplets (LDs) compared to normal cells [12], which might promote the proliferation process, thereby favoring the development of a more aggressive phenotype [13]. Moreover, evidence indicates that a higher level of LDs in cancer cells is associated with higher tumor aggressiveness [14, 15]. Recently, LDs showed a powerful effect on cellular lipid metabolism, which regulates lipid synthesis, lipolysis, and lipid turnover [16, 17]. The proteins decorated in the monolayer of phospholipids of LDs control the activities of LDs [18–20]. Therefore, the LD-related proteins are involved in the regulation of cellular lipid metabolism.

The ubiquitin–proteasome system (UPS) is a huge and complex system that regulates the degradation of up to 80% of cellular proteins, consisting of ubiquitin (Ub), Ub-activating enzymes (E1s, two classes, nearly 10 members), Ub-conjugating enzymes (E2s, approximately 40 members), Ub-protein ligases (E3s, more than 600 members), deubiquitinating enzymes (DUBs, roughly 90 members), and the heart of the system, the 26S proteasome complex [21–25]. Previous studies showed that UPS is involved in a wide range of biological processes, including cell growth, the cell cycle, DNA metabolism, and signal transduction [26, 27]. The deregulation of UPS or its components results in severe pathological disorders and changes in the expression levels of many tumor promoters and suppressors [21, 28–31]. The 26S proteasome is a multi-enzyme complex that responds to the degradation of intracellular proteins in eukaryotic cells [32–34]. *PSMD1* and *PSMD2* are two subunits of the 19S regulatory complex of the 26S proteasome [35–38]. Recent studies indicate that knockdown of *PSMD1* and/or *PSMD2* is able to suppress tumor cell proliferation [39–41]. Many studies about the proteomes of LDs have found that *PSMD1* and *PSMD2* are LD-related proteins in several species such as humans, mice, and *C. elegans* [42–48]. However, the regulatory roles of *PSMD1* and *PSMD2* in cellular lipid metabolism are unclear.

In the present study, we chose a hepatocellular carcinoma cell line, HepG2, to investigate the roles of *PSMD1 *and *PSMD2* in cell proliferation and cellular lipid metabolism. HepG2 cells were derived from 15-year-old white liver cancer tissue and were utilized in the study about hepatocyte metabolism. Firstly, we showed that the expression levels of *PSMD1* and *PSMD2* affected cell proliferation and apoptosis. The knockdown of *PSMD1* and *PSMD2* inhibited cell proliferation and promoted cell apoptosis, and overexpression of *PSMD1* and *PSMD2* showed the opposite effects. Furthermore, the expression of *PSMD1* and *PSMD2* affected several proliferation and apoptosis related molecules. Because cellular lipid content is associated with cell proliferation and apoptosis, we further investigated the effects of *PSMD1* and *PSMD2* expression on cellular lipid metabolism. The knockdown of *PSMD1* and *PSMD2* decreased the content of cellular lipids. On the contrary, the overexpression of *PSMD1* and *PSMD*2 promoted lipid formation and increased the cellular lipid content. Mechanically, *PSMD1* and *PSMD2* inhibition suppressed fatty acid and lipid synthesis by downregulating *SREBF1* and *PPARγ *via ASK-p38-JNK and AKT signaling. Moreover, through bioinformatic analysis, high expression levels of *PSMD1* and *PSMD*2 were significantly correlated with poor prognosis of liver hepatocellular carcinoma. Therefore, the high expression levels of *PSMD1* and *PSMD2* probably enhanced hepatocellular carcinoma tumor cell proliferation. *PSMD1* and *PSMD2* could be potential therapeutic targets for this type of tumor.

## Materials and methods

### Cell culture and transfection

HepG2 and Huh7 cells were purchased from the Type Culture Collection of the Chinese Academy of Sciences (Wuhan, China). Cells were cultured with Dulbecco’s modified Eagle’s medium (DMEM, HyClone, Logan, UT, USA) supplemented with 10% fetal bovine serum (FBS, AusGeneX, Molendinar, Australia) at 37 °C in a humidified atmosphere of 5% CO_2_. Cells were transfected with Lipo8000™ Transfection Reagent (#C0533, Beyotime, Nanjing, China). HepG2 cells were seeded on the cell slide in a 6-well plate and transfected with the plasmid vector in accordance with the transfection reagent instructions.

### Oleic acid treatment

For oleic acid treatment, a 20 mM oleic acid (LPS free)-phosphate buffer saline (PBS) mixture and 20% FA-free bovine serum albumin (BSA) medium were prepared, and both media were heated in a 70 °C water bath for 30 min. Finally, the media were mixed. The 10 mM oleic acid–BSA mixture was added to the cell cultural medium at 1:49 (v:v). To identify the best treatment time, a time course was performed. The cells were treated with 200 μM oleic acid (OA) for 0, 1, 2, 3, 4, 5, and 6 h respectively, and then were stained by BODIPY to indicate the cellular LDs. The images showed that cellular LDs were able to be observed at 2 h after OA treatment. Many LDs formed “grape-like” structures at 5 h after OA treatment, and additionally, the image from the 6 h treatment showed little difference from the image from the 5 h treatment (Additional file 1: Fig. S1A). Therefore, the 6 h treatment was considered to be the best choice for the experiment. The cells that were seeded on the slides or plates were washed 3 times using PBS. One millilitre of oleic acid medium was added to the well (200 μM), and the cells were cultured for 6 h.

### MTT assay

HepG2 cell proliferation was detected using MTT Cell Proliferation and Cytotoxicity Assay Kit (Beyotime, Nanjing, China). Briefly, cells were seeded at a density of 4 × 10^3^/well in 96-well plates 24 h after transfection. At different time points, 10 μL MTT (5 mg/mL) was added to each well and cells were cultured for another 4 h. Furthermore, 100 μL Formazan solution was added into each well and the optical density (OD) at 570 nm was measured on a microplate reader (PerkinElmer, Germany).

### CCK8 assay

HepG2 and Huh7 cells activity after oleic acid treatment was detected by Cell Counting Kit-8 (CCK8) kit (#CK04, DOJINDO LABORATORISE, Shanghai, China). Briefly, cells were seeded at a density of 4 × 10^3^/well in 96-well plates. The cells were treated with 200 μM oleic acid for 0 h, 6 h, 12 h and 24 h. Then 10 μL CCK8 was added to each well and cells were cultured for another 4 h. The optical density (OD) at 450 nm was measured on a microplate reader (PerkinElmer, Germany).

### Flow cytometric analysis of the cell cycle

Cells were collected and fixed in 70% ice-cold ethanol at 4 °C overnight. Then, the cells were centrifuged and washed with PBS. After incubation with 100 μL RNase A (0.1 mg/mL) for 30 min at 37 °C, 2 μL propidium iodide (PI, Sigma-Aldrich, St. Louis, MO, USA) was add to the cells and incubated for another 30 min at room temperature in the dark. Finally, cell cycle profiling was performed using a FACSCalibur Flow Cytometer (Becton–Dickinson, Franklin Lakes, NJ, USA), and the data were analyzed using ModFit software (Verity Software House).

### Cell apoptosis assay

At 48 h after transfection, the percentage of apoptotic cells was measured using an Annexin V fluorescein isothiocyanate (FITC) apoptosis detection kit (BD Biosciences, USA) and analyzed by flow cytometry (BD Biosciences, USA) in accordance with the manufacturer’s protocol.

### 5-Ethynyl-2′-deoxyuridine (EdU) assay

Cells seeded in 12-well plates were cultured to 50% confluence and then transfected. Forty-eight hours after transfection with siRNAs or overexpression plasmid, the cells were fixed and stained with a BeyoClick™ EdU Cell Proliferation Kit with Alexa Fluor 594 (Beyotime, China) in accordance with the instructions. A fluorescence microscope (Olympus, Japan) was used to capture three randomly selected fields to visualize the number of EdU-positive cells.

### RNA oligonucleotides and plasmid construction

siRNA oligos against *PSMD*1 and *PSMD2* were obtained from Ribobio (Guangzhou, China). The sequence of the siRNA against the *PSMD1* coding region was 5′-GAGAAAGACAGUGACUCGAUGGAAA-3′, and that of the siRNA against the *PSMD2* coding region was 5′- CCCAAGGUGCCUGAUGACAUCUACA-3′ [49]. The expression vector used in this study was constructed according to the instructions of a Seamless Cloning Kit (Vazyme Biotech Co., Ltd.). The primers used for plasmid vector construction are listed in Additional file 2: Table S1.

### RNA interference and overexpression assay

The siRNAs targeting *PSMD1* and *PSMD2* were obtained from Ribobio (Guangzhou, China). Cells were seeded onto 6-well plates in DMEM supplemented with 10% FBS at 37 °C in a humidified atmosphere of 5% CO_2_. Cells at 70–80% confluence were transfected with 10 µL of siRNA using Lipo8000™ Transfection Reagent (#C0533, Beyotime, Nanjing, China). For the overexpression assay, 4 µg of the constructed plasmid was transfected with 10 µL of Lipo8000™ Transfection Reagent (#C0533, Beyotime, Nanjing, China). All transfections were performed in triplicate, and the data are presented as the mean ± standard deviation (SD).

### RNA extraction, reverse transcription polymerase chain reaction (RT-PCR), and real-time quantitative RT-PCR (qRT-PCR)

Total RNA was extracted using TRIzol (Life Technologies Inc., Carlsbad, CA, USA). RNA concentrations were determined using spectrophotometry with a NanoDrop 2000 (Thermo Fisher Scientific). A total of 1 μg of RNA was then subjected to reverse transcription to cDNA using a Reverse Transcription Kit (#R232-01; HiScript II Q Select RT SuperMix for qPCR; Vazyme, Nanjing, China). qRT-PCR was performed with SYBR Green I Real-Time PCR Master Mix (#Q131-02; AceQ qPCR SYBR Green Master Mix; Vazyme; Nanjing, China) on a LightCycler^®^ 480 Real-Time System (Roche) and QuantStudio 6 Flex Real-Time PCR System (ABI, Thermo Fisher, Shanghai, China). The primer sequences used for qRT-PCR are listed in Additional file 2: Table S2. *GAPDH* and *ACTB*, the most frequently used housekeeping genes, were chosen as candidates. Then, the stability of the candidate reference genes was analyzed through standard curve and geNorm methods (Additional file 2: Tables S3, S4). Consequently, *GAPDH* was chosen as the reference standard for normalization. The related method was based on that used in Costantini et al.’s study [50]. The relative gene expression was calculated by the 2^−ΔΔ^Ct method, and the Student’s *t* test was used for statistical analysis.

### Western blotting

Western blotting was performed as reported previously [51]. The following antibodies were used: anti-*PSMD1* (#A16420; rabbit polyclonal antibody; ABclonal; 1:1000 dilution), anti-*PSMD2* (#14748-1-AP; rabbit polyclonal antibody; Proteintech; 1:1000 dilution), anti-ASK1 (#A6274; rabbit polyclonal antibody; ABclonal; 1:2000 dilution), anti- p-ASK1 (#AP0394; rabbit polyclonal antibody; ABclonal; 1:2000 dilution), anti-AKT (#A11027; rabbit polyclonal antibody; ABclonal; 1:2000 dilution), anti-p-AKT (#AP0140; rabbit polyclonal antibody; ABclonal; 1:2000 dilution), anti-p38 (#A10832; rabbit polyclonal antibody; ABclonal; 1:2000 dilution), anti-p-p38 (#AP0297; rabbit polyclonal antibody; ABclonal; 1:2000 dilution), anti-JNK1/2 (#A11119; rabbit polyclonal antibody; ABclonal; 1:2000 dilution), anti-p-JNK1/2 (#AP0631; rabbit polyclonal antibody; ABclonal; 1:2000 dilution), anti-GAPDH (Servicebio, Wuhan, China; 1:2000 dilution), anti-Tubulin (Servicebio, Wuhan, China; 1:2000 dilution), HRP-labeled Goat Anti-Rabbit IgG (H + L) (#GB23303-1, Servicebio, Wuhan, China), and HRP-labeled Goat Anti-Mouse IgG (H + L) (#GB23301, Servicebio, Wuhan, China).

### Lipid droplet marking and observation

Cells were seeded on slides on a 24-well plate and were cultured for 24 h. The slides were fixed in 4% paraformaldehyde (Servicebio, Wuhan, China) for 15 min at room temperature. The slides were stained with BODIPY 493/503 (#D3922, Invitrogen, Carlsbad, CA, USA) for 45 min at 37 °C and were then stained with DAPI (#G-1012, Servicebio) for 10 min at 37 °C. After washing three times with PBS for 10 min each, the slides were sealed with an anti-fluorescent quenching solution (#P36961, ProLong™ Diamond Antifade Mountant, Invitrogen™, Carlsbad, CA, USA) for use in a confocal laser scanning microscope (Zeiss LSM 800, Carl Zeiss, German) observation.

### Co-localization of *PSMD1*/*PSMD2* and LDs

The fluorescence labelling *PSMD1*, *PSMD2*, and PLIN2 expression vectors (*PSMD1*-mCherry, *PSMD2*-mCherry and PLIN2-EGFP) were co-transfected into HepG2 cells. After 48 h, the cells were treated with 200 μM OA for another 6 h. Then, the cells were fixed and then sealed with an anti-fluorescent quenching solution for confocal laser scanning microscope observation. The fluorescence plot analysis was performed by ImageJ software according to the instructions (https://imagej.en.softonic.com/). For the co-localization of *PSMD1*/*PSMD2* and newly formed LDs, Livedrop was introduced to this experiment. The Livedrop [52] has been reported to be a useful fluorescence marker to label the newly formed LDs from the ER. The newly formed LDs were detected by Livedrop-EGFP within 30 min. Then, the same experiment was performed. In our study, we detected that the newly formed LDs were 45 min old.

### Survival analysis and normal/cancer gene expression comparison analysis

Survival predication was performed using the GEPIA database (http://gepia.cancer-pku.cn/). The prognosis analysis and gene expression analysis were performed according to the instructions of the creator of this database [53].

### Statistical analysis

All experiments were repeated at least three times. All results are presented as the mean ± SD. Statistical significance was assessed using Student’s t-test. A p value < 0.05 was deemed to indicate statistical significance.

## Results

### Both *PSMD1* and *PSMD2* knockdown suppresses HepG2 cell proliferation but promotes apoptosis

**Fig. 1:**
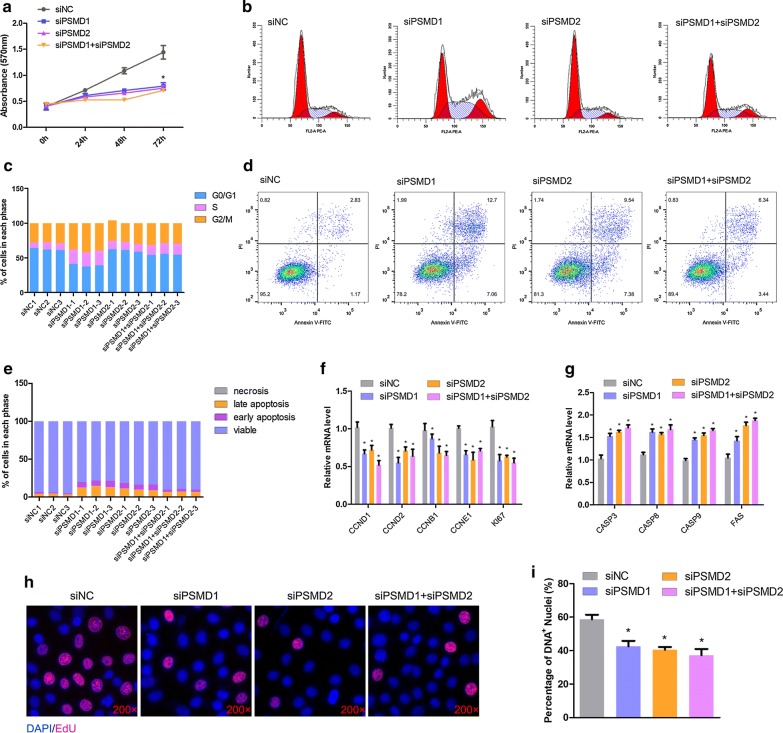
*PSMD1* and *PSMD2* knockdown inhibits proliferation and induces cell apoptosis. **a** Cell proliferation was monitored with an MTT assay at the indicated time after treatment with *PSMD1* and *PSMD2* siRNAs in HepG2 cells. Data are presented as the mean ± SD (n = 3), *p < 0.05. **b**, **c** Flow cytometric analysis of the cell cycle distribution at 48 h post-transfection of the negative control (NC) or *PSMD1*/*PSMD2* siRNA in HepG2 cells. The percentages of each phase of the cell cycle (G0/G1, S, and G2/M) are shown. Data are presented as the mean ± SD (n = 3), *p < 0.05, **p < 0.01. **d**, **e** HepG2 cells were collected for the detection of apoptotic cells by flow cytometry, 48 h after transfection. In all panels, data are presented as the mean ± SEM of three independent assays. The statistical significance of differences between means was assessed using an unpaired Student’s t-test (*p < 0.05; **p < 0.01) vs. the NC. **f** Relative mRNA expression of the cell cycle-related genes after transfection of siPSMD1/siPSMD2 and siNC. Independent sample t-tests were used to analyze the statistical differences between groups. *p < 0.05; **p < 0.01. **g** The mRNA expression levels of several apoptosis-related genes with siPSMD1/siPSMD2 and siNC in HepG2 cells. Independent sample t-tests were used to analyze the statistical differences between groups. *p < 0.05; **p < 0.01. **h**, **i** EdU staining after PSMD knockdown. The magnification is ×200. Results are shown as the mean ± SEM of three independent experiments. Independent sample t-tests were used to analysis the statistical differences between groups. *p < 0.05; **p < 0.01

The idea that *PSMD1* and *PSMD2* play important roles in the proliferation process of tumor cells, such as breast cancer cells and lung cancer cells, is not novel. However, few studies have reported their roles in regulating the proliferation and apoptosis process in HepG2 cells. Thus, we first investigated the functions of *PSMD1* and *PSMD2* in HepG2 cells. The specific RNAi fragments were prepared to block the expression of *PSMD1* and *PSMD2*—individually and together. The cell proliferation capacity was detected by the MTT method, indicating that the cell proliferation process was significantly inhibited by the downregulation of *PSMD1* and *PSMD2* (p < 0.05, Fig. 1a). We then performed a cell cycle analysis to illustrate the cells in different phases. The results indicated that more cells were blocked in the S-stage by *PSMD1* and *PSMD2* knockdown (Fig. 1b, c). Then, the apoptosis analysis was performed through the flow cytometry method. As expected, more cells underwent apoptosis with low expression levels of *PSMD1* and *PSMD2* (Fig. 1d, e). To investigate the potential regulatory mechanism, we detected the expression levels of proliferation-related (Fig. 1f) and apoptosis-related genes (Fig. 1g). The expression levels of *CCND1*, *CCND2*, *CCNB1*, *CCNE1,* and *ki67* were downregulated significantly (p < 0.05) in the siPSMD1, siPSMD2, and siPSMD1 + siPSMD2 group cells, while *CASP3*, *CASP8*, *CASP9,* and *FAS* were upregulated significantly (p < 0.05). To validate this result, the EdU assay was performed to detected cell proliferation. The number of proliferating cells was approximately 60% of the total cells in the control group, whereas the percentages were about 40%, 39%, and 38% in the siPSMD1, siPSMD2, and siPSMD1 + siPSMD2 groups, respectively (Fig. 1h, i). Taken together, *PSMD1* and *PSMD2* played important roles in the cell proliferation and apoptosis processes.

### *PSMD2* and *PSMD2* overexpression promotes HepG2 cell proliferation but suppresses apoptosis

**Fig. 2:**
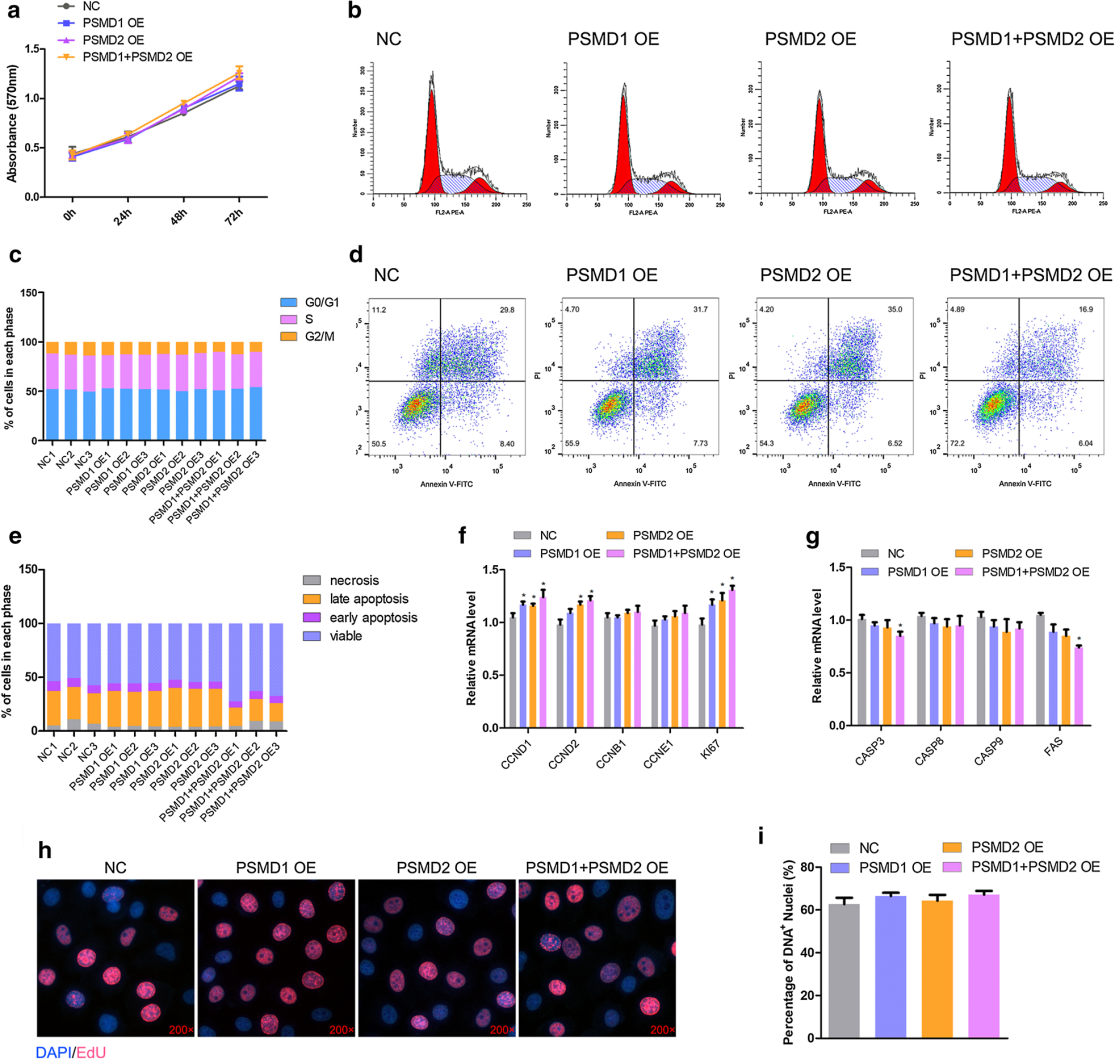
*PSMD1* and *PSMD2* overexpression promotes proliferation and inhibits cell apoptosis. **a** Cell proliferation was monitored with the MTT assay at the indicated time after treatment with *PSMD1*/*PSMD2* overexpression in HepG2 cells. Data are presented as the mean ± SD (n = 3), *p < 0.05. **b**, **c** Flow cytometric analysis of the cell cycle distribution at 48 h post-transfection of overexpression plasmid in HepG2 cells. The percentages of each phase of the cell cycle (G0/G1, S, and G2/M) are shown. **d**, **e** HepG2 cells were collected for the detection of apoptotic cells by flow cytometry, 48 h after transfection. In all panels, data are presented as the mean ± SEM of three independent assays. **f** Relative mRNA expression of the cell cycle-related genes after transfection of the *PSMD1*/*PSMD2* plasmid. The statistical significance of differences between means was assessed using unpaired Student’s t-tests (*p < 0.05; **p < 0.01) vs. the NC. **g** The mRNA expression levels of several apoptosis-related genes with the overexpression plasmid in HepG2 cells. The statistical significance of differences between means was assessed using unpaired Student’s t-tests (*p < 0.05; **p < 0.01) vs. the NC. **h**, **i** EdU staining after *PSMD1*/*PSMD2* overexpression. The magnification is 200×. Results are shown as the mean ± SEM of three independent experiments. Independent sample t-tests were used to analyze the statistical differences between groups. *p < 0.05; **p < 0.01

To further investigate the functions of *PSMD1* and *PSMD2* in cell proliferation, *PSMD1* and *PSMD2* were overexpressed individually or together. The MTT assay indicated that *PSMD1* and *PSMD2* overexpression increased the cell proliferation capacity slightly but not significantly (Fig. 2a). We then performed the cell cycle analysis. There were no significant changes among different groups of cells (Fig. 2b, c). Subsequently, the apoptosis analysis was performed, and interestingly, the number of cells undergoing apoptosis increased only in groups with both *PSMD1* and *PSMD2* overexpression (Fig. 2d, e). Therefore, we detected the expression levels of genes related to proliferation (Fig. 2f) and apoptosis (Fig. 2g). Interestingly, the expression levels of *CCND1*, *CCND2,* and *ki67* increased slightly but significantly (p < 0.05, Fig. 2f), while the expression levels of *CASP3* and *FAS* decreased in the *PSMD1* OE + *PSMD2* OE group (Fig. 2g). To validate the result, the EdU assay was performed to detect the number of proliferating cells. There were no significant differences observed among the different treatment groups (Fig. 2h, i).

### *PSMD1* and *PSMD2* knockdown inhibits the formation of cellular lipid droplets

**Fig. 3:**
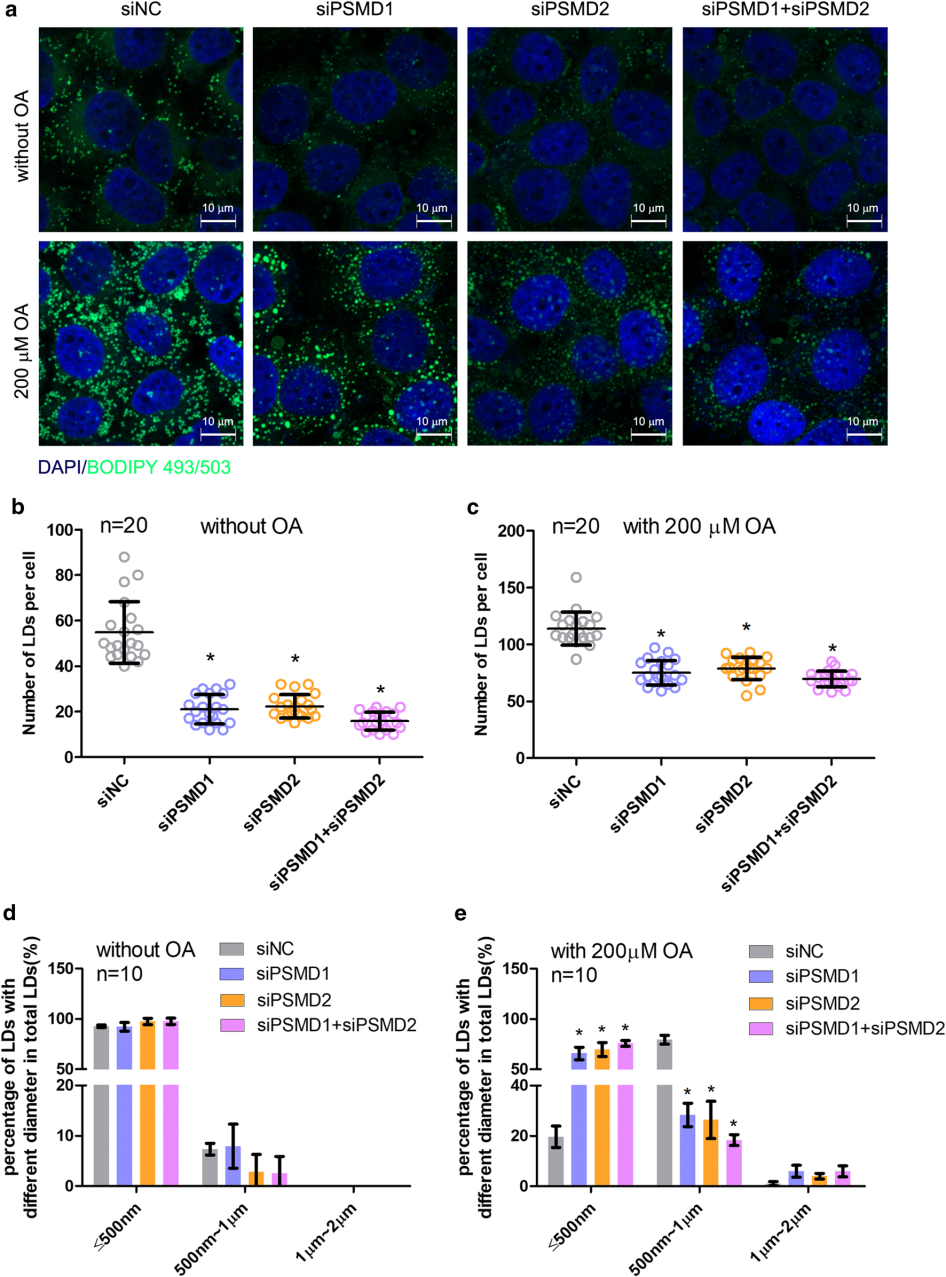
*PSMD1* and *PSMD2* knockdown regulates the number and size of cellular lipid droplets. **a** Detection of the cellular LDs marked by BODIPY493/503 through a laser scanning confocal microscope. The HepG2 cells were seeded on the slide in a 24-well plate. Then, the cells were transfected with *PSMD1/PSMD2* or NC siRNAs for 48 h. Subsequently, the cells were treated with 200 μM oleic acid for another 6 h. Then, the cells were fixed and stained by BODIPY493/503 and DAPI for observation by microscope. **b**, **c** The number of cellular LDs of different groups of cells in the absence or present of OA. ImageJ software was used for the analysis. The statistical significance of differences between means was assessed using an unpaired Student’s t-test (n = 20; *p < 0.05; **p < 0.01) vs. NC. **d**, **e** The count of the size of cellular LDs of different groups of cells in the absence or present of OA. ImageJ software was used for the analysis. Statistical significance of differences between means was assessed using an unpaired Student’s t-test (n = 10; *p < 0.05; **p < 0.01) vs. NC

Cellular lipid metabolism is important for cell proliferation and apoptosis. We investigated the effects of *PSMD1* and *PSMD2* on the formation of lipid droplets (LDs). Usually, cells form the fatty acid and lipid droplets de novo. The cellular LDs were marked by BODIPY493/503, a specific neutral lipid targeting dye, and the numbers and sizes of LDs in different cell groups were analyzed based on the images captured by the confocal laser scanning microscope (Fig. 3a). The LD number in the siPSMD1, siPSMD2, and siPSMD1 + siPSMD2 cell groups was significantly less than the number of control group cells (p < 0.05, Fig. 3b). Furthermore, we detected the capacity of neutral lipid synthesis of different group cells by treating them with 200 μM oleic acid (OA) for 12 h. Before OA treatment experiment, we detected the toxicity of OA on both HepG2 cells and Huh7 cells by utilizing CCK8 method. The results showed that OA treatment showed less toxicity on these two cell lines with the treatment time of 6 h, 12 h and 24 h (Additional file 1: Fig. S1C, D). Subsequently, we found that siPSMD1, siPSMD2, and both interfered cell groups contained fewer LDs than the control cells (p < 0.05, Fig. 3c). Then, we calculated the LD size and found that *PSMD1* and *PSMD2* interference did not change the LD size in the absence of OA, whereas the suppression of *PSMD1* and *PSMD2* increased the diameters of LDs in FA-rich medium (Fig. 3d, e). To further validate the regulatory roles of *PSMD1* and *PSMD2* in lipid metabolism, we performed the same experiments using another human hepatocellular carcinoma cell line, Huh7. We found that the knockdown of *PSMD1* and/or *PSMD2* decreased the cellular LD number significantly (p < 0.05, Additional file 3: Fig. S2A–C).

### *PSMD1* and *PSMD2* overexpression promotes the formation of lipid droplets

**Fig. 4:**
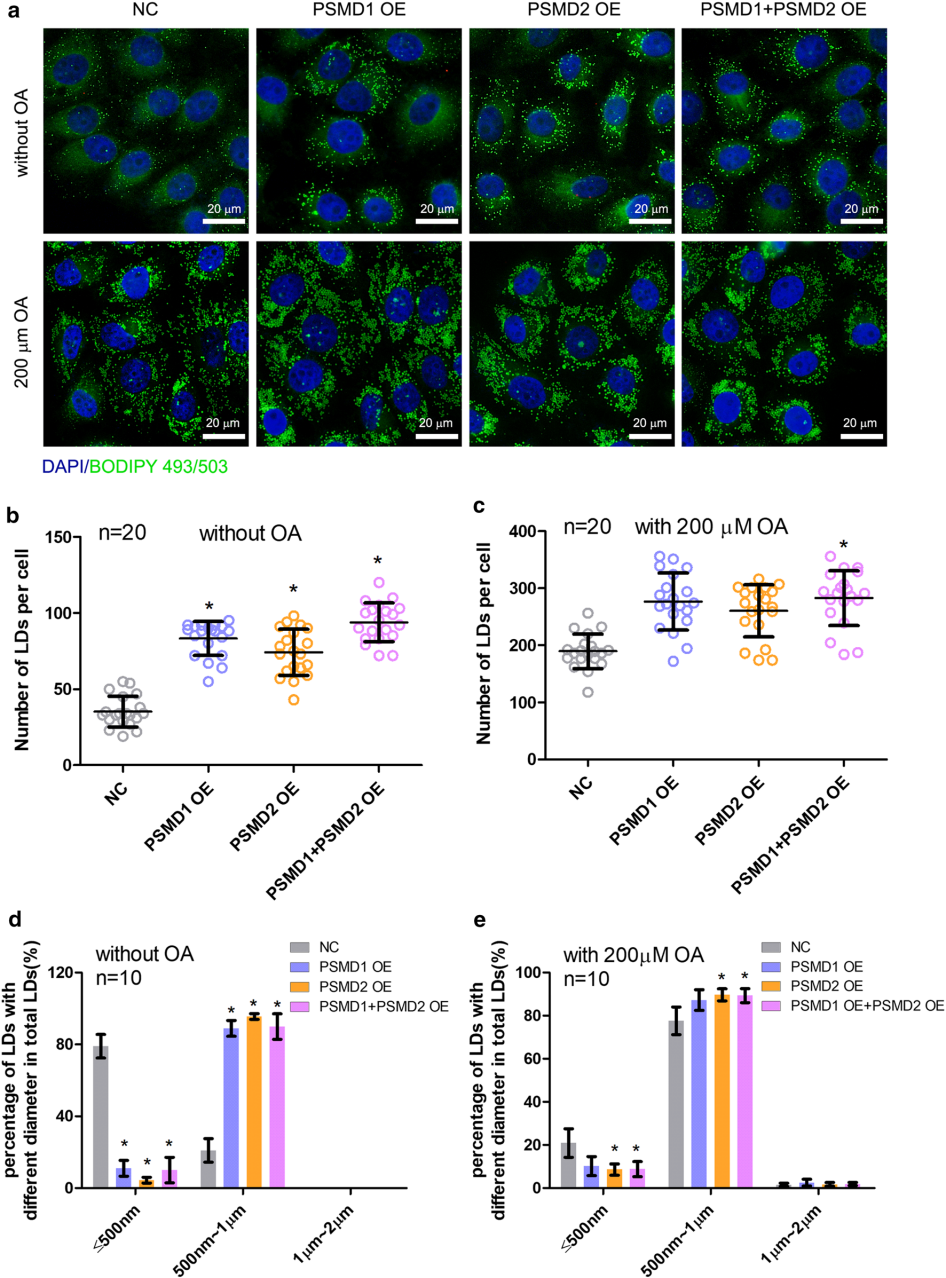
*PSMD1* and *PSMD2* overexpression regulates the number and size of cellular lipid droplets. **a** Detection of the cellular LDs marked by BODIPY493/503 through a laser scanning confocal microscope. The HepG2 cells were seeded on the slide in a 24-well plate. Then, the cells were transfected with *PSMD1/PSMD2* or NC overexpression plasmid for 48 h. Subsequently, the cells were treated with 200 μM oleic acid for another 6 h. Then, the cells were fixed and stained by BODIPY493/503 and DAPI for observation by microscope. **b**, **c** The number of cellular LDs from different groups of cells in the absence or present of OA. ImageJ software was used for the analysis. The statistical significance of differences between means was assessed using an unpaired Student’s t-test (n = 20; *p < 0.05; **p < 0.01) vs. NC. **d**, **e** The size of cellular LDs of different groups of cells in the absence or present of OA. ImageJ software was used for the analysis. The statistical significance of differences between means was assessed using an unpaired Student’s t-test (n = 10; *p < 0.05; **p < 0.01) vs. NC

To further investigate the effects of *PSMD1* and *PSMD2* on cellular LD formation and growth, we overexpressed these two genes in HepG2 cells and detected the LD number and size by fluorescence labeling of LDs. In both the absence and presence of OA, *PSMD1* and *PSMD2* overexpression significantly increased the number of cellular LDs compared with that of control cells (p < 0.05, Fig. 4a–c). Interestingly, larger LDs were observed in cells with *PSMD1* and/or *PSMD2* overexpression compared with control cells (Fig. 4a, d), whereas the difference in LD size between the overexpression and control group cells was decreased and almost disappeared in the presence of OA (Fig. 4e), although the overexpression group cells contained sporadic larger LDs with diameters between 1 and 2 μm (Fig. 4a, e). For further validation, the Huh7 cells were utilized to detect whether overexpression of *PSMD1* and/or *PSMD2* affected the cellular LD number. We found that the overexpression of *PSMD1* and/or *PSMD2* increased the LD diameter in Huh7 cells (Additional file 3: Fig. S2D–F). The phenotypes seen in the overexpression experiment contrasted with the results seen in the interference experiment, and therefore, *PSMD1* and *PSMD2* were potential positive regulators in the cellular lipid formation process.

### *PSMD1* and *PSMD2* regulate the expression level of FA and lipid synthesis-related genes

**Fig. 5:**
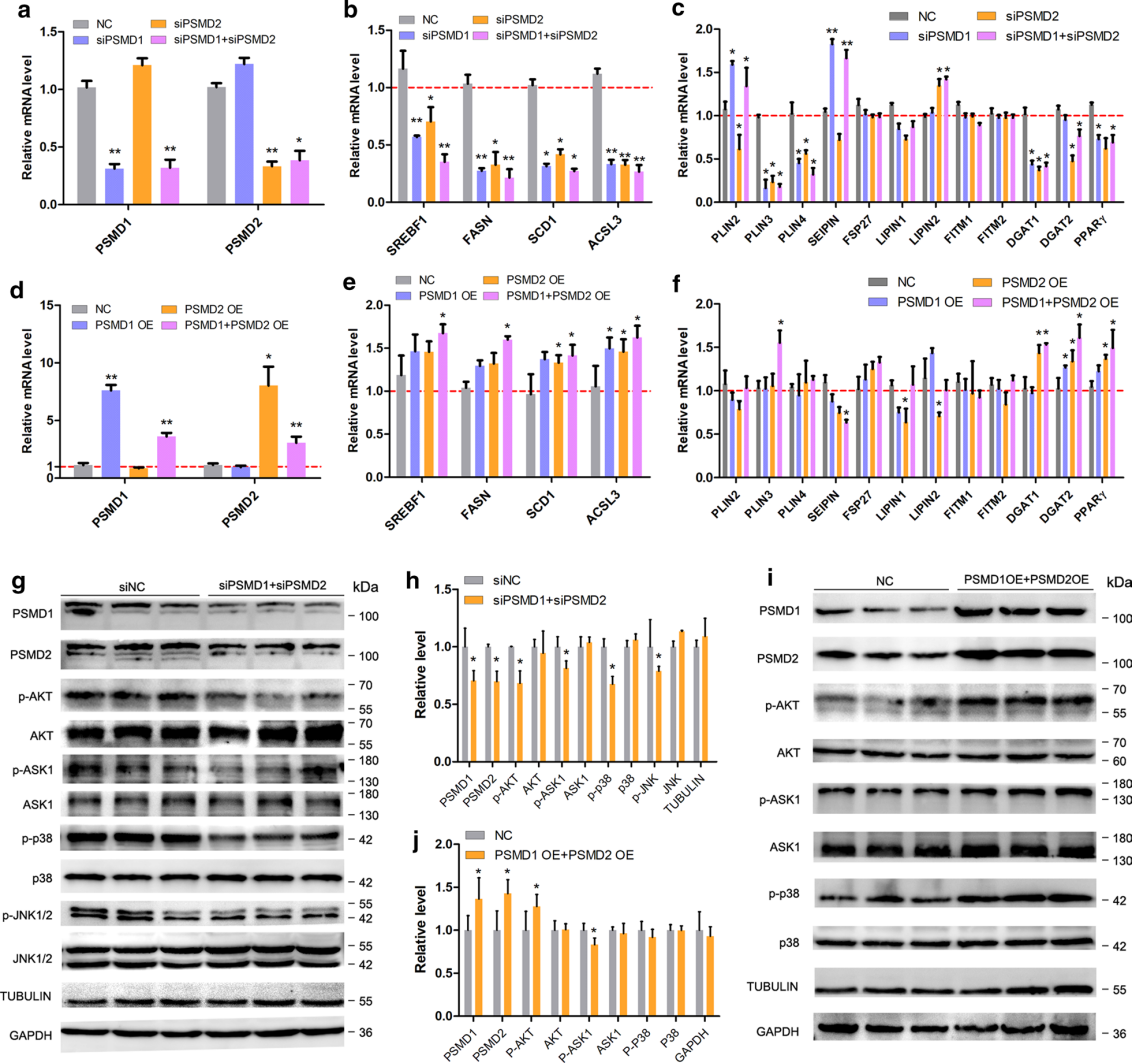
*PSMD1* and *PSMD2* regulate the expression level of fatty acids (FAs) and lipid synthesis-related genes. **a** Interference efficiency detection by qRT-PCR. **b** The expression level of fatty acid synthesis-related genes was detected by qRT-PCR. **c** The expression level of lipid synthesis-related genes was detected by qRT-PCR. **d** Overexpression efficiency detection by qRT-PCR. **e** The expression level of fatty acid synthesis-related genes was detected by qRT-PCR. **f** The expression level of lipid synthesis-related genes was detected by qRT-PCR. **g** The ASK1-p38-JNK and AKT signaling in groups of interfered cells and control cells was detected by Western Blot experiments. TUBULIN and GAPDH were the reference proteins. **h** Grey value analysis of **g**. ImageJ software was used for this analysis, according to the instructions. **i** The ASK1-p38-JNK and AKT signaling in the overexpression cell group and control cells was detected by Western Blot experiments. TUBULIN and GAPDH were the reference proteins. **j** Grey value analysis of **i**. ImageJ software was used for this analysis, according to the instructions. The statistical significance of differences between means was assessed using an unpaired Student’s t-test (n = 3; *p < 0.05; **p < 0.01) vs. NC

The changes in LD number and size prompted us to investigate the effects of *PSMD1* and *PSMD2* on the expression levels of lipid metabolic-related genes including the FA-related genes (*SREBF1*, *FASN*, *SCD1,* and *ACSL3*) and neutral lipid synthesis-related genes (*PLINs*, *LIPINs*, *FITMs*, *DGATs*, *PPAR*γ, *SEIPIN,* and *FSP27*). We first investigated the effect of the suppression of *PSMD1* and *PSMD2* expression on these genes. The efficiency of interference was reflected by the mRNA levels of *PSMD1* and *PSMD2* detected by qPCR (Fig. 5a), and the expression levels of *PSMD1* and *PSMD2* decreased by approximately 70%. Then, the expression levels of FA and lipid synthesis-related genes were detected. As expected, the expression levels of *SREBF1*, *FASN*, *SCD1,* and *ACSL3* were significantly decreased in the interference group cells compared with the control cells (p < 0.05, Fig. 5b), which suggests that the de novo FA synthesis capacity was decreased in the interference group cells. Moreover, the expression of neutral lipid synthesis-related genes, such as *DGAT1*, *DGAT2* and *PPARγ*, was decreased by the suppression of *PSMD1* and/or *PSMD2* (p < 0.05, Fig. 5c). Interestingly, we found that *PSMD1* and *PSMD2* showed different effects on the expression of *PLIN2*, a well-known LD marker gene. *PSMD1* interference promoted the expression of *PLIN2*, whereas the *PSMD2* interference decreased *PLIN2* expression. Additionally, *PSMD1* interference increased the expression of *SEIPIN*, also known as *BSCL2*, which plays an important role in cellular LD formation and budding off, whereas *PSMD2* interference did not change the expression of SEIPIN. Subsequently, we also investigated the effects of *PSMD1* and/or *PSMD2* overexpression on the expression of these genes. *PSMD1* and *PSMD2* were highly overexpressed (Fig. 5d). The expression of *SREBF1*, *FASN*, *SCD1,* and *ACSL3* was significantly increased in the *PSMD1* OE + *PSMD2* OE cell groups (p < 0.05, Fig. 5e). Furthermore, the expression levels of *DGAT1*, *DGAT2,* and *PPARγ* were significantly upregulated by *PSMD1* and *PSMD2* overexpression (p < 0.05, Fig. 5f). It was interesting that the expression level of *SEIPIN* was downregulated by *PSMD1 *+ *PSMD2* OE (p < 0.05), which was in contrast to the results of the interference experiment. This result suggests that *PSMD1* and *PSMD2* may regulate *SEIPIN* expression.

To investigate the molecular mechanism by which *PSMD1* and *PSMD2* regulate FA and lipid synthesis-related genes, we detected two classical pathways, the ASK1-p38-JNK and AKT signaling pathways. We detected the phosphorylated ASK1, p38, and JNK1/2 levels and found that the p-ASK1, p-p38, and p-JNK1/2 levels were decreased in interference group cells compared with control cells (Fig. 5g, h). Additionally, the p-AKT level was also decreased in the interference group cells compared with the control cells (Fig. 5g, h). Subsequently, we investigated the effects of *PSMD1* and *PSMD2* overexpression on these two signaling pathways. As expected, the p-AKT and p-ASK1 levels increased significantly but mildly (p < 0.05, Fig. 5i, j). Additionally, the p-p38 level was not significantly different (Fig. 5i, j). Although the effects of *PSMD1* and *PSDM2* overexpression on ASK1 and AKT signaling were not obvious, we also considered that *PSMD1* and *PSMD2* regulated these two signaling pathways, thereby regulating FA and neutral lipid synthesis.

### *PSMD1* and *PSMD2* regulate cellular lipid metabolism

**Fig. 6:**
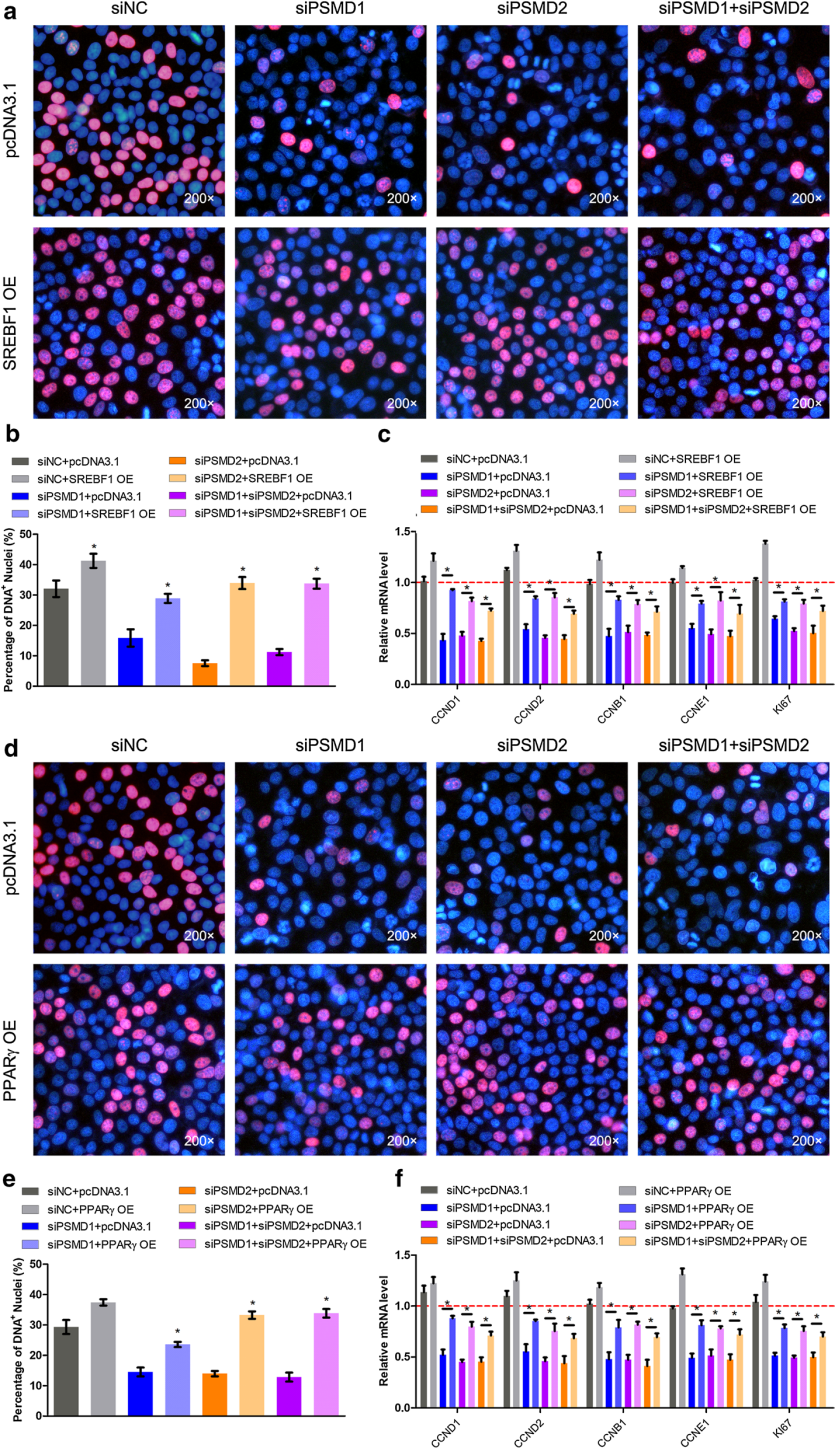
*PSMD1* and *PSMD2* regulate cellular lipid metabolism. HepG2 cells were seeded in the 6-well plate. Then, the cells were transfected with *PSMD1*/*PSMD2* siRNAs or *SREBF1*/*PPARγ* overexpression vectors or control siRNA or a control overexpression vector. **a**, **d** Cell proliferation capacity was monitored by the EdU assay. **b**, **e** Statistical analysis was performed on the EdU assay. The magnification is 200×. Results are shown as the mean ± SEM of three independent experiments. Independent sample t-tests were used to analyze the statistical differences between groups. *p < 0.05; **p < 0.01. **c**, **f** Relative mRNA expression of the cell-cycle-related genes. Independent sample t-tests were used to analysis the statistical differences between groups (n = 3), *p < 0.05; **p < 0.01

To validate that *PSMD1* and *PSMD2* regulate cell proliferation through modulating cellular FA and lipid content, we performed a rescue experiment that overexpressed *SREBF1* or *PPARγ* in the *PSMD1* and *PSMD2* knockdown cells. We showed that *PSMD1* and *PSMD2* knockdown inhibited cell proliferation. Subsequently, we tried to increase the FA level by overexpressing *SREBF1* in *PSMD1* and *PSMD2* knockdown cells, because *SREBF1* could promote the transcription of FA-synthesis-related enzymes. Then, the capacity of cell proliferation was detected by EdU assay. The results showed that *SREBF1* overexpression increased the number of proliferating cells in the siPSMD1, siPSMD2, and siPSMD1 + siPSMD2 groups compared with the corresponding control groups (p < 0.05, Fig. 6a, b). Moreover, the expression levels of proliferation-related genes were also detected by qPCR. Although *SREBF1* overexpression did not recover the proliferation impaired by *PSMD1* and/or *PSMD2* interference totally, the proliferation capacity did increase compared with that of the control (Fig. 6b, c). Furthermore, *PPARγ* overexpression increased the proliferation capacity of *PSMD1* and/or *PSMD2* knockdown cells (Fig. 6d, e). Additionally, the expression levels of proliferation-related genes, including *CCNs* and *ki67,* increased compared with that of the corresponding control group (p < 0.05, Fig. 6f). These two rescue experiments indicated that increasing the lipid content in cells could partly recover the *PSMD1* and *PSMD2* interference-induced inhibition of proliferation. The results showed that *PSMD1* and *PSMD2* can affect cell proliferation by regulating cellular lipid metabolism.

### *PSMD1* and *PSMD2* localize to the surface of lipid droplets

**Fig. 7:**
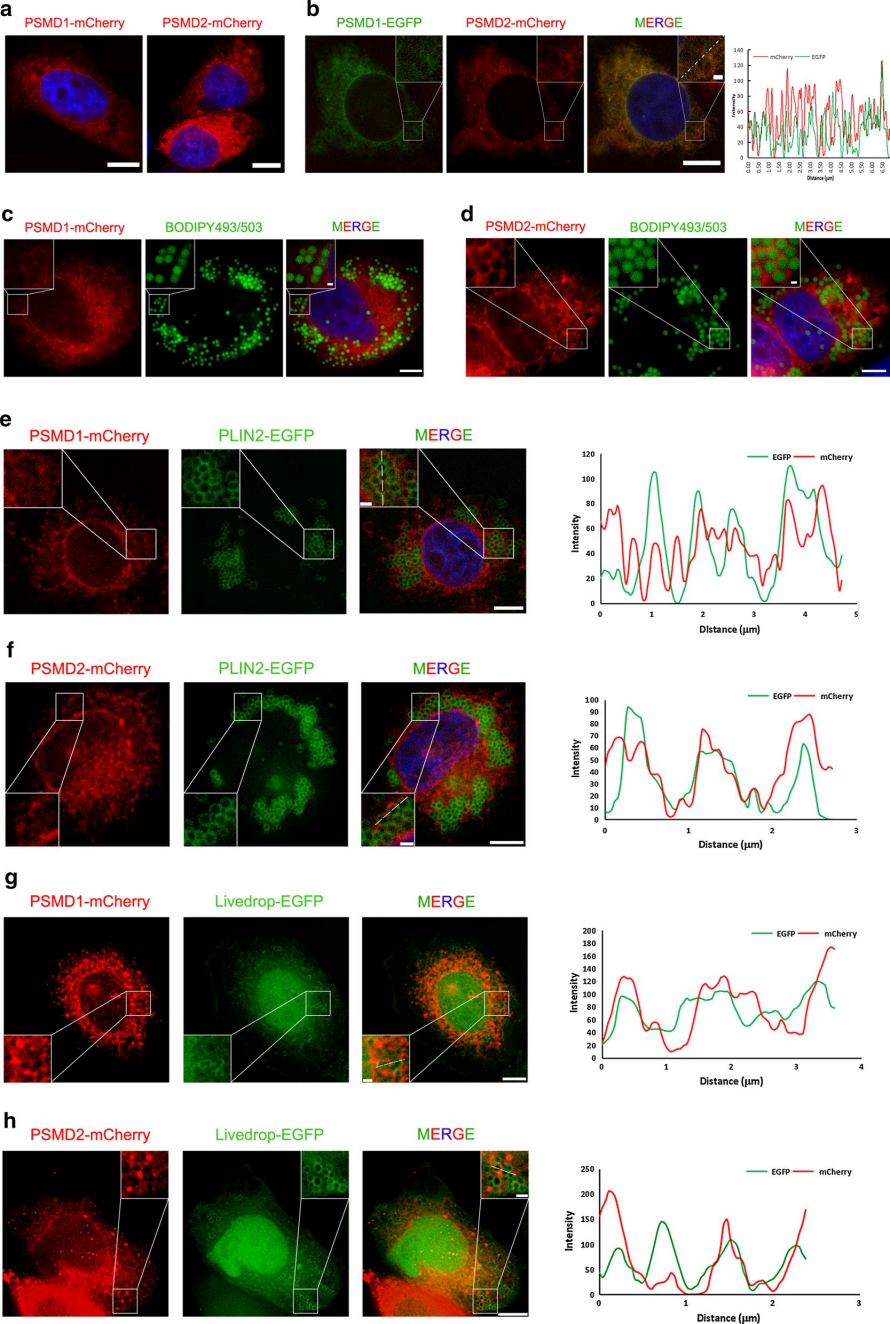
*PSMD1* and *PSMD2* localize to the surface of lipid droplets. Fluorescence expression vectors were constructed, including *PSMD1*-mCherry, *PSMD2*-mCherry, *PSMD1*-EGFP, PLIN2-EGFP, and Livedrop-EGFP. **a** *PSMD1*-mCherry or *PSMD2*-mCherry was transfected into cells for 48 h. Then, the cells were fixed and stained by DAPI. The images were captured by laser scanning confocal microscope (bar = 10 μm). **b** *PSMD1*-EGFP and *PSMD2*-mCherry were co-transfected into cells for 48 h. Then, the cells were fixed and stained by DAPI. The images were captured by a laser scanning confocal microscope (bar = 10 μm). The fluorescence plot was analyzed by ImageJ software. **c**, **d** The cells were transfected with *PSMD1*-mCherry or *PSMD2*-mCherry for 48 h, and then the cells were treated with 200 μM oleic acid (OA) for another 6 h. Then, the cells were fixed and stained by BODIPY493/503 and DAPI. The images were captured by a laser scanning confocal microscope (bar = 10 μm). **e**, **f** The cells were co-transfected with *PSMD1*-mCherry or *PSMD2*-mCherry and PLIN2-EGFP vectors for 48 h, and then the cells were treated with 200 μM OA for another 6 h. Then, the cells were fixed and stained by BODIPY493/503 and DAPI. The images were captured by laser scanning confocal microscope (bar = 10 μm). **g**, **h** The cells were co-transfected with *PSMD1*-mCherry or *PSMD2*-mCherry and Livedrop-EGFP vectors for 48 h, and then the cells were treated with 200 μM OA for another 6 h. Then, the cells were fixed and stained by BODIPY493/503 and DAPI. The images were captured by a laser scanning confocal microscope (bar = 10 μm)

We detected the subcellular localization of *PSMD1* and *PSDM2* and the co-localization of *PSMD* and LDs. *PSMD1* and *PSMD2* are the components of the 19S subunit of proteasome; therefore, they are mainly distributed in the cytoplasm. We labelled *PSMD1* and *PSMD2* by the mCherry-tag in their C-terminals, and their distribution was captured by a confocal laser scanning microscope (Fig. 7a). As expected, *PSMD1* and *PSMD2* were localized in the cytoplasm (Fig. 7a). Then, we labelled *PSMD1* by an EGFP-tag in its C-terminal to determine the co-localization of *PSMD1* and *PSMD2*. The results showed that *PSMD1* and *PSMD2* share the same localization (Fig. 7b). Furthermore, we investigated the co-localization of *PSMD1*/*PSMD2* and cellular LDs. The signals of *PSMD1*-mCherry/*PSMD2*-mCherry and BODIPY493/503 were captured and the images of Fig. 7c, d showed that *PSMD1*/*PSMD2* were localized to the outside of the LDs. To validate the *PSMD1*/*PSMD2* localization accurately, the PLIN2-EGFP was co-transfected with the *PSMD1*-mCherry or *PSMD2*-mCherry expression vector, because PLIN2 is a well-known LD marker localized to the surface of LDs. By fluorescence distribution analysis, the signals of *PSMD1*/*PSMD2* were almost overlapped with the signal of PLIN2 (Fig. 7e, f). It should be noted that *PSMD1*/*PSMD2* are not strict peri-LD proteins, because we did not observe the “round-ring” signal like we did for PLIN2. According to these results, we conclude that *PSMD1* and *PSMD2* are able to localize to cellular LDs, which could be the potential method by which *PSMD1* and *PSMD2* regulate cellular LD formation and growth.

It is well known that LDs are generated from the endoplasmic reticulum (ER). The LDs can carry proteins from the ER and recruit proteins through the ARF-COPI system. Therefore, we further investigated the source of LD localized *PSMD1*/*PSMD2* proteins. We detected the signals of Livedrop-EGFP and *PSMD1*-mCherry/*PSMD2*-mCherry at 45 min after OA treatment. The fluorescence analysis showed that two signals shared a high level of overlap, which indicated that *PSMD1* and *PSMD2* had localized to the newly formed LDs (Fig. 7g, h). This result suggests that LDs might carry *PSMD1* and *PSMD2* from the ER during their generation.

### High expression of *PSMD1* and *PSMD2* is associated with the poor prognosis of liver hepatocellular carcinomas

**Fig. 8:**
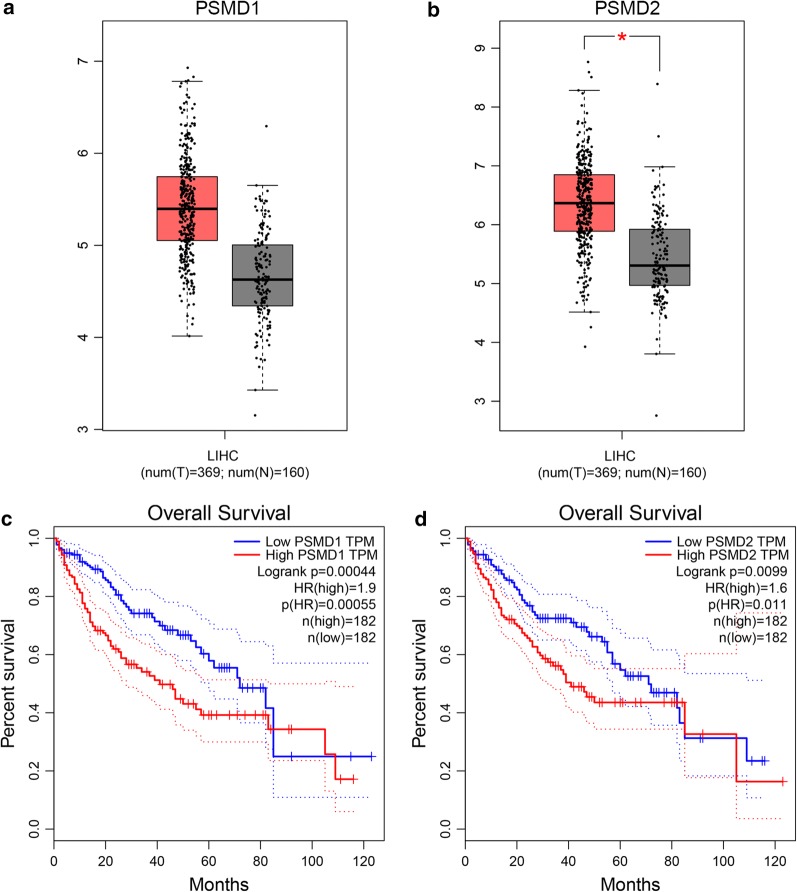
*PSMD1* and *PSMD2* show higher expression in liver hepatocellular carcinoma (LIHC) and are associated with a poor prognosis. Gene expression analysis and survival analysis were performed by the GEPIA (gene expression profiling interactive analysis) database (http://gepia.cancer-pku.cn/). **a**, **b** Box plot of expression of *PSMD1* and *PSMD2* in LIHC. **c**, **d** The survival analysis of *PSMD1* and *PSMD2* in LIHC

To investigate the effect of high expression of *PSMD1* or *PSMD2* on the prognosis of liver hepatocellular carcinoma (LIHC), we analyzed the expression levels of *PSMD1* and *PSMD2* in LIHC via the GEPIA (gene expression profiling interactive analysis) database (http://gepia.cancer-pku.cn/). The data showed that *PSMD1* and *PSMD2* are highly expressed in LIHC (Fig. 8a, b). Furthermore, the results showed that LIHC with high expression of *PSMD1* and *PSMD2* is associated with a poor prognosis (Fig. 8c, d).

## Discussion

In the present study, we identified a novel way in which *PSMD1* and *PSMD2* promote tumor cell proliferation—namely, via increasing cellular FA and lipid synthesis. It is not novel that the proteasome can regulate the proliferation and apoptosis of tumor cells [54, 55], because proteasomes are associated with the degradation of the majority of cellular proteins. Therefore, as the important subunits of the 26S proteasome, *PSMD1* and *PSMD2* also regulate tumor cell growth. Many previous studies also have reported their function in cell proliferation. For example, *PSMD1* could regulate breast cancer cell growth [41], and *PSMD2* can regulate tumor cell proliferation in lung cancer and breast cancer [39, 56]. Furthermore, *PSMD2* reduces proliferation and induces apoptosis in lung cancer cells, and the overexpression of *PSMD2* could stimulate cell growth in SMMC-7721 and NIH-3T3 cells [40, 57]. However, the way in which *PSMD1* and *PSMD2* regulate tumor cell proliferation is not totally clear. Previous studies demonstrated that *PSMD1* or *PSMD2* regulate the expression levels of p53, p21, and p27 [39–41], which are cell cycle and cell growth regulators. Our findings provide new insight into the regulatory roles of *PSMD1* and *PSMD2* in tumor cell proliferation via regulating lipid metabolism.

Recently, lipid metabolism was found to be important for the proliferation and growth of tumor cells. Cells need many kinds of lipids to support their duplication and growth, and they also require enough FAs for necessary biological processes. Cellular FAs and lipids are usually stored in LDs, novel organelles that have been known about for in decades, which play important roles in many biological processes such as lipid formation and degradation, cell proliferation and apoptosis, anti-stimulation, and cellular immunity [19, 58–63]. Recently, researchers found the LDs play an important role in tumor carcinogenesis and metastasis. Aboumrad et al. identified that a high number of lipid vesicles in the cytoplasm is a characteristic of mammary carcinoma [64]. Additionally, Ramos et al. reported that high lipid-containing mammary carcinoma has more aggressive behavior [6]. Lipid-rich characterization has become a clinically distinctive form of carcinoma [6, 65]. A high level of LDs is associated with higher tumor aggressiveness [15] and chemotherapy resistance [14], and additionally, tumorigenesis-related proteins can be recruited and stored in LDs [66–68], such as PI3K, ERK1, and ERK2. Tumor cells can regulate the LD content via the EGFR–PI3K–mTOR and FOXO/SIRT6 pathways, and LDs also regulate the proliferation and growth of tumor cells via membrane lipid and energy formation [69–74], which establishes a tight association between LDs and tumor cells.

It is very interesting that *PSMD1* and *PSMD2* can regulate the cellular LD content. As far as we know, no study has reported this regulatory function of *PSMD1* and *PSMD2* in tumor cells. It should be noted that the idea that the proteasome pathway regulates LD formation, stabilization, and degradation is not novel, and there are many studies about it. For example, the proteasome inhibitor MG132 can suppress the degradation of ADRP (also known as PLIN2) [75], and additionally, a proteasome-dependent pathway can regulate the level of ADRP to modulate the cellular TG content [76]. Moreover, Doa10, an ER-associated degradation ubiquitin ligase, is able to regulate the levels of some LD proteins [77], and additionally, the ubiquitin ligases and Spartin/SPG20 can regulate the number and size of LDs [78]. Furthermore, *SIRT7* controls hepatic lipid formation and accumulation via regulating the ubiquitin–proteasome pathway, and *SIRT7* knockout mice have shown resistance to high-fat-diet induced fatty liver [79]. Moreover, a previous functional genomic screen study in Drosophila S2 cells suggested that proteasomes are involved in the regulation of cellular lipids [80]. All of these studies demonstrated that UPS is essential for LD biology. However, ubiquitin–proteasome is a huge system, which consists of numerous components. The functions and regulatory mechanisms of these components are poorly understood. In this study, we focused on the two important subunits, *PSMD1* and *PSMD2*, which could affect the cellular lipid content. We detected the FA and lipid synthesis enzyme expression levels during *PSMD1/PSMD2* knockdown or cell overexpression, and the results indicated that *PSMD1* and *PSMD2* indeed affect the expression levels of *FASN*, *SCD1*, *SREBF1*, *DGAT,* and *PPARγ* (Fig. 5). Although some changes in expression were mild, significant changes in the LD number can be observed in Figs. 3a and 4a (p < 0.05), indicating that the capacity of cellular FA and de novo lipid synthesis indeed changed. Subsequently, we also investigated the pathways impacted by *PSMD1* and *PSMD2*. Previous studies showed that ASK1-p38-JNK signaling regulates the expression level of *SREBF1* and the activation of SREBP1c, which could regulate the de novo cellular FA synthesis process [81–85]. Additionally, AKT signaling is important for cellular metabolism as it regulates cell proliferation and differentiation [86–93]. We found that *PSMD1/PSMD2* knockdown impaired ASK1 and AKT signaling, which indicates that cellular lipid formation and energy metabolism were suppressed. This genotype corresponds to the phenotype where cell proliferation was inhibited and apoptosis was promoted (Fig. 1). On the other hand, *PSMD1/PSMD2* overexpression enhanced ASK1 and AKT signaling, although the changes were mild (Fig. 5i, j), which corresponds to the phenotype where the cell-proliferation-related genes were upregulated and cell apoptosis was decreased (Fig. 2). For this phenotype, we considered that *PSMD1* and *PSMD2* are medium to highly expressed genes, so overexpression might not lead to obvious effects on cellular proliferation, and furthermore, previous studies have reported that the overexpression of *PSMD2* could stimulate cell growth [40, 57]. Therefore, there is no doubt that *PSMD1* and *PSMD2* could enhance the cell proliferation process. We then tried to validate the idea that *PSMD1/PSMD2* regulate cell proliferation via regulating lipid metabolism. Since *PSMD1/PSMD2* knockdown downregulated the expression of FA and lipid synthesis genes, we recovered their expression by transfection with *SREBF1* or *PPARγ* expression vectors. These two genes are upstream transcriptional factors of FA and lipid synthesis enzymes. The results indicated that cell proliferation was recovered partly when the cells were transfected with *SREBF1* or *PPARγ* (Fig. 6), which suggests that this treatment was able to compensate for the impairment of *PSMD1/PSMD2* knockdown on cell proliferation. We then investigated the co-localization of *PSMD1*/*PSMD2* and LDs. Both the mature LDs (labelled by PLIN2-EGFP) and newly formed LDs (labelled by Livedrop-EGFP) were found to be related to the localization of *PSMD1* and *PSMD2* (Fig. 7f–h), which indicated that *PSMD1*/*PSMD2* were localized to LDs during the generation progression. Moreover, the localization of *PSMD1*/*PSMD2* on the LD surface suggests that proteasomes might degrade the proteins recruited on LD surfaces. However, the significance of the localization of *PSMD1* and *PSMD2* on LD surfaces still requires further study. A prognosis analysis was performed to illustrate the association between the *PSMD1* and *PSMD2* expression levels and survival rates. The results indicated that high expression levels of *PSMD1* and *PSMD2* decrease the survival rate of LIHC (Fig. 8c, d), which corresponds to our study where *PSMD1* and *PSMD2* increased the LD content and promoted tumor cell proliferation.

## Conclusion

**Fig. 9:**
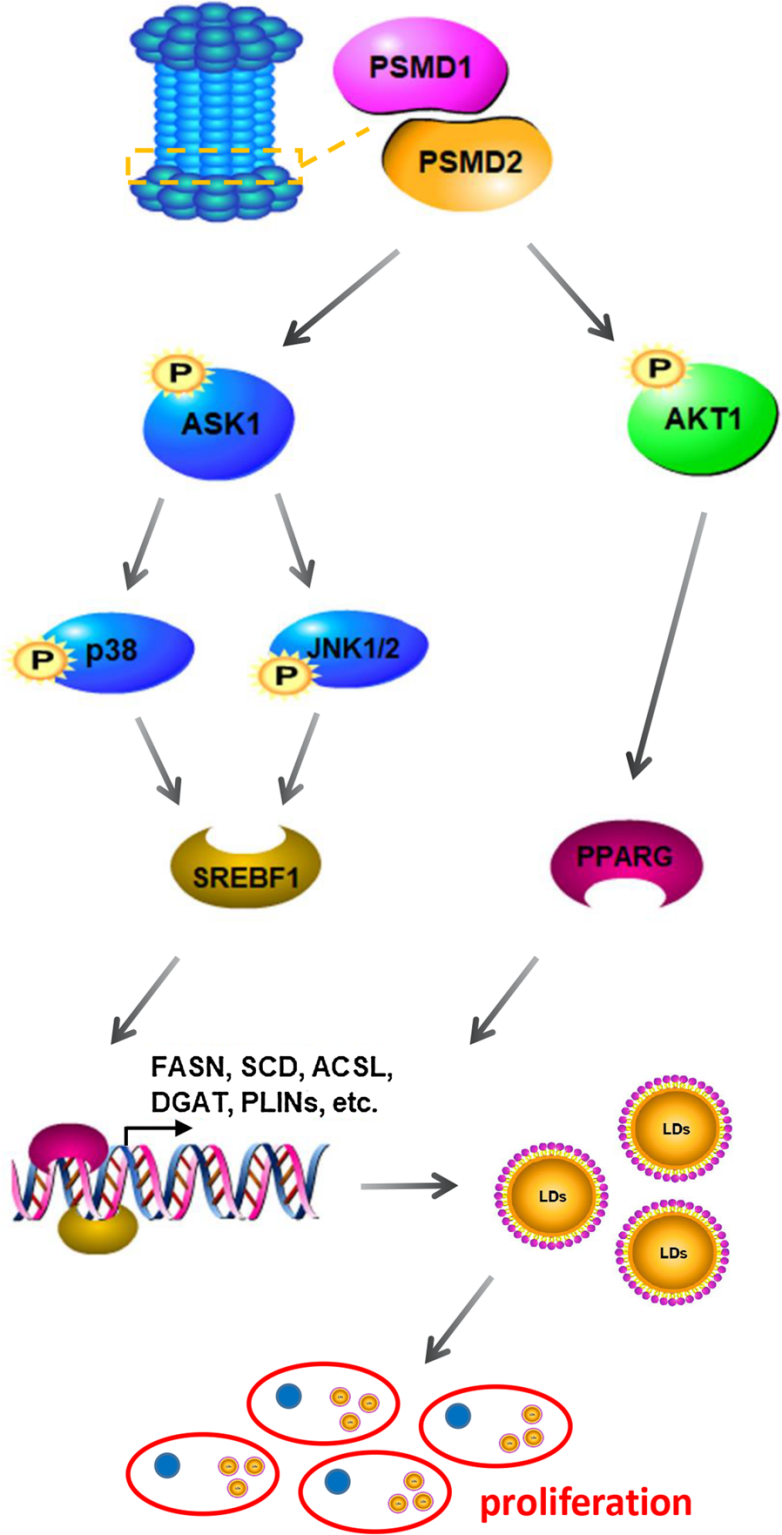
Overview of the regulatory mechanism of *PSMD1* and *PSMD2* that regulates tumor cell proliferation. Briefly, *PSMD1* and *PSMD2* regulate the phosphorylation of ASK1-p38-JNK and AKT signaling. Then, the expression level of *SREBF1* and *PPARγ* is regulated. Subsequently, these two important transcriptional factors regulate the expression levels of FAs and lipid synthesis-related genes such as *FASN, SCD1, ASCL, DGAT* and *PLIN*. The upregulation of these genes induces more lipid droplet (LD) formation, whereas LDs can provide membrane components and energy for cell proliferation. Therefore, *PSMD1* and *PSMD2* regulate cell proliferation via modulating cellular lipid metabolism

In the present study, we identified that *PSMD1* and *PSMD2* could regulate the cellular lipid content, thereby affecting cell proliferation progression. Mechanistically, *PSMD1* and *PSMD2* regulate FA and lipid synthesis-related gene expression through ASK1-p38-JNK and AKT signaling. The molecular mechanism of *PSMD1*/*PSMD2* regulating cell proliferation is illustrated in Fig. 9. This study provides new insights into the *PSMD1*/*PSMD2* regulatory mechanism in HCC cell proliferation and provides a potential novel therapeutic strategy for lipid-rich tumor.

## Supplementary information

**Additional file 1: Fig. S1.** Time course experiment of oleic acid medium treatment. **A** The cells were treatment with 200 μM oleic acid. The cellular lipid droplets were imaged at 1 h, 2 h, 3 h, 4 h, 5 h and 6 h after oleic acid treatment. **B, C** The cell activity of Huh7 cells and HepG2 cells was detected by CCK8 method after 200 mM oleic acid medium treatment for 6 h, 12 h and 24 h.

**Additional file 2.** Additional tables.

**Additional file 3: Fig. S2.** *PSMD1* and *PSMD2* expression level regulates the number and size of cellular lipid droplets in Huh7 cells. The Huh7 cells were seeded on the slide in a 24-well plate. Then, the cells were transfected with *PSMD1*/*PSMD2* or NC siRNAs for 48 h for knockdown (**A**–**C**), or transfected with *PSMD1*/*PSMD2* expression vector or NC vector for 48 h for overexpression (**D**–**F**). Subsequently, the cells were treated with 200 μM oleic acid for another 6 h. Then, the cells were fixed and stained by BODIPY493/503 and DAPI for observation by microscope. (**B**, **E**) The number of cellular LDs from different groups of cells. ImageJ software was used for the analysis. The statistical significance of differences between means was assessed using an unpaired Student’s t-test (n = 20; *p < 0.05) vs. NC. (**C**, **F**) The size of cellular LDs of different groups of cells. ImageJ software was used for the analysis. The statistical significance of differences between means was assessed using an unpaired Student’s t-test (n = 10; *p < 0.05;) vs. NC.

## Data Availability

The original data of the real-time PCR experiments, images for western blot analysis, images for fluorescence analysis will be available upon request.

## Abbreviations

*PSMD1*: proteasome 26S subunit, non-ATPase 1
*PSMD2*: proteasome 26S subunit, non-ATPase 2
UPS: ubiquitin proteasome system
E1: Ub-activating enzyme
E2: Ub-conjugating enzyme
E3: Ub-protein ligase
DUB: deubiquitinating enzyme
PLIN1: perilipin1
PLIN2: perilipin2
PLIN3: perilipin3
PLIN4: perilipin4
PLIN5: perilipin5
FSP27/CIDEC: cell death-inducing DFFA-like effector c
*SREBF1*: sterol regulatory element binding transcription factor 1
*PPARγ*: peroxisome proliferator activated receptor gamma
JNK/MAPK8: mitogen-activated protein kinase 8
OA: oleic acid
SEIPIN/BSCL2: seipin lipid droplet biogenesis associated
FITM1: fat storage inducing transmembrane protein 1
FITM2: fat storage inducing transmembrane protein 2
ATGL/PNPLA2: patatin like phospholipase domain containing 2
CCND1: cyclin D1
CCND2: cyclin D2
CCNB1: cyclin B1
CCNE1: cyclin E1
Ki67: marker of proliferation Ki-67
CASP3: caspase3
CASP8: caspase8
CASP9: caspase9
FAS: Fas cell surface death receptor
FASN: fatty acid synthase
SCD1: stearoyl-CoA desaturase 1
ACSL3: acyl-CoA synthetase long chain family member 3
DGAT1: diacylglycerol O-acyltransferase 1
DGAT2: diacylglycerol O-acyltransferase 2
ASK1/MAP3K5: mitogen-activated protein kinase kinase kinase 5
LIHC: liver hepatocellular carcinoma
