# Supplementary figures and images for: PSMD1 and PSMD2 regulate HepG2 cell proliferation and apoptosis via modulating cellular lipid droplet metabolism
Source: BMC Mol Biol. 2019 Nov 8;20:24. doi: 10.1186/s12867-019-0141-z (PMC6842266; doi:10.1186/s12867-019-0141-z)

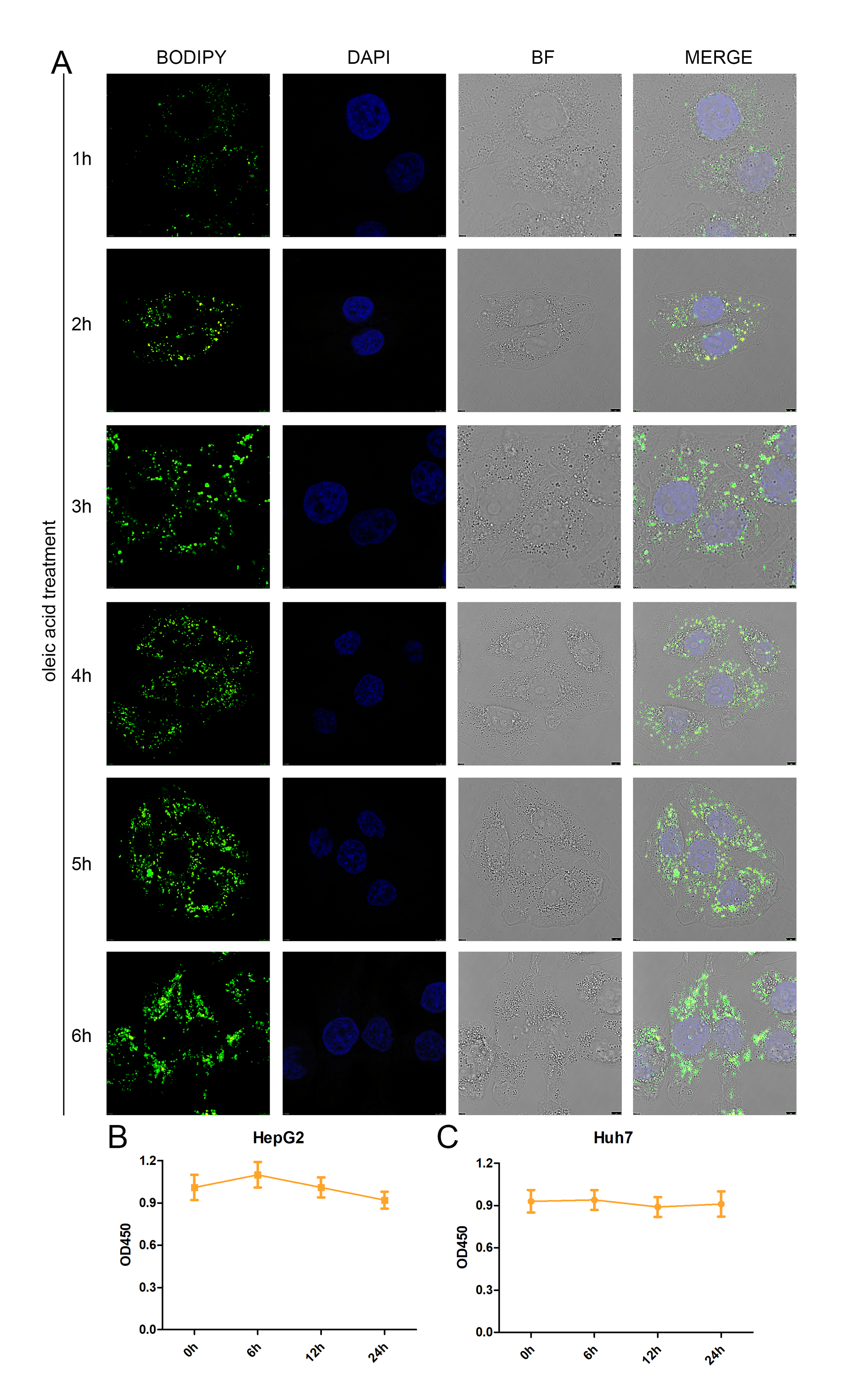

Supplement: Supplementary file 1 — Additional file 1: Fig. S1. Time course experiment of oleic acid medium treatment. A The cells were treatment with 200 μM oleic acid. The cellular lipid droplets were imaged at 1 h, 2 h, 3 h, 4 h, 5 h and 6 h after oleic acid treatment. B, C The cell activity of Huh7 cells and HepG2 cells was detected by CCK8 method after 200 mM oleic acid medium treatment for 6 h, 12 h and 24 h. [file 12867_2019_141_MOESM1_ESM.tif]

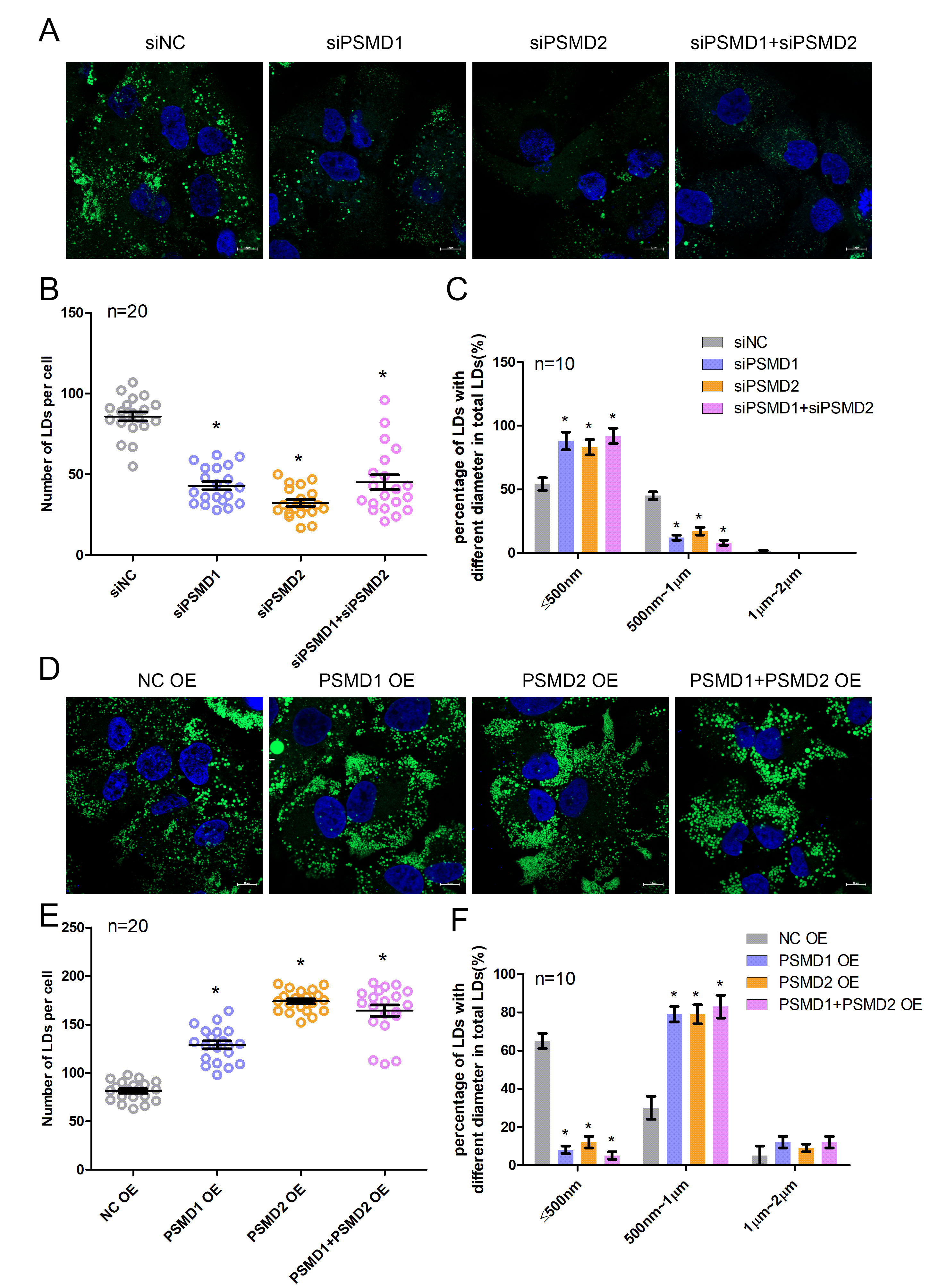

Supplement: Supplementary file 3 — Additional file 3: Fig. S2. PSMD1 and PSMD2 expression level regulates the number and size of cellular lipid droplets in Huh7 cells. The Huh7 cells were seeded on the slide in a 24-well plate. Then, the cells were transfected with PSMD1/PSMD2 or NC siRNAs for 48 h for knockdown (A–C), or transfected with PSMD1/PSMD2 expression vector or NC vector for 48 h for overexpression (D–F). Subsequently, the cells were treated with 200 μM oleic acid for another 6 h. Then, the cells were fixed and stained by BODIPY493/503 and DAPI for observation by microscope. (B, E) The number of cellular LDs from different groups of cells. ImageJ software was used for the analysis. The statistical significance of differences between means was assessed using an unpaired Student’s t-test (n = 20; *p < 0.05) vs. NC. (C, F) The size of cellular LDs of different groups of cells. ImageJ software was used for the analysis. The statistical significance of differences between means was assessed using an unpaired Student’s t-test (n = 10; *p < 0.05;) vs. NC. [file 12867_2019_141_MOESM3_ESM.tif]
