# Supplementary material for: PSMD1 and PSMD2 regulate HepG2 cell proliferation and apoptosis via modulating cellular lipid droplet metabolism
Source: BMC Mol Biol. 2019 Nov 8;20:24. doi: 10.1186/s12867-019-0141-z (PMC6842266; doi:10.1186/s12867-019-0141-z)
Supplement: Supplementary file 2 — Additional file 2. Additional tables. [file 12867_2019_141_MOESM2_ESM.docx]

## Additional Tables

### Table S1: Primers used in the overexpression experiment.

| **primer** | **Sequences** (5’-3’) |
| --- | --- |

PSMD1-F CDS ctagcgtttaaacttaagcttATGATCACCTCGGCCGCT

PSMD1-R CDS ccacactggactagtggatccTTAATCATCAATATACTCAAATGGTTCTG

PSMD2-F CDS ctagcgtttaaacttaagcttATGATCACCTCGGCCGCT

PSMD2-R CDS ccacactggactagtggatccTTAATCATCAATATACTCAAATGGTTCTG

PPARG-F CDS ctagcgtttaaacttaagcttATGACCATGGTTGACACAGAGATG

PPARG-R CDS ccacactggactagtggatccCTAGTACAAGTCCTTGTAGATCTCCTGC

SREBF1-F CDS ctagcgtttaaacttaagcttATGGACGAGCCACCCTTCA

SREBF1-R CDS ccacactggactagtggatccCTAGCTGGAAGTGACAGTGGTCC

### Table S2: Sequences of PCR primers used in this study.

| **primer** | **Sequences** (5’-3’) |
| --- | --- |
| PSMD1-F  PSMD1-R  PSMD2-F  PSMD2-R  CCND1-F  CCND1-R  CCND2-F  CCND2-R  CCNB1-F  CCNB1-R  CCNE1-F  CCNE1-R  KI67-F  KI67-R  CASP3-F  CASP3-R  CASP8-F  CASP8-R  CASP9-F  CASP9-R  FAS-F  FAS-R  SREBF1-F  SREBF1-R  FASN-F  FASN-R  SCD1-F  SCD1-R  ACSL3-F  ACSL3-R  PLIN2-F  PLIN2-R  PLIN3-F  PLIN3-R  PLIN4-F  PLIN4-R  SEIPIN-F  SEIPIN-R  FSP27-F  FSP27-R  LIPIN1-F  LIPIN1-R  LIPIN2-F  LIPIN2-R  FITM1-F  FITM1-R  FITM2-F  FITM2-R  DGAT1-F  DGAT1-R  DGAT2-F  DGAT2-R  PPARG-F  PPARG-R | TGTTGGCACCCCTATTGCTTC  TGTTTCCATCGAGTCACTGTCT  TGCTCGTGGAACGACTAGG  CAGTTTGCCATAGTGTGGACG  GCTGCGAAGTGGAAACCATC  CCTCCTTCTGCACACATTTGAA  TTTGCCATGTACCCACCGTC  AGGGCATCACAAGTGAGCG  AATAAGGCGAAGATCAACATGGC  TTTGTTACCAATGTCCCCAAGAG  GCCAGCCTTGGGACAATAATG  CTTGCACGTTGAGTTTGGGT  ACGCCTGGTTACTATCAAAAGG  CAGACCCATTTACTTGTGTTGGA  CATGGAAGCGAATCAATGGACT  CTGTACCAGACCGAGATGTCA  TTTCTGCCTACAGGGTCATGC  TGTCCAACTTTCCTTCTCCCA  CTCAGACCAGAGATTCGCAAAC  GCATTTCCCCTCAAACTCTCAA  AGATTGTGTGATGAAGGACATGG  TGTTGCTGGTGAGTGTGCATT  CGGAACCATCTTGGCAACAGT  CGCTTCTCAATGGCGTTGT  AAGGACCTGTCTAGGTTTGATGC  TGGCTTCATAGGTGACTTCCA  TCTAGCTCCTATACCACCACCA  TCGTCTCCAACTTATCTCCTCC  ATGGAAAACCAACCTCATAGCAA  GCCATCCCAGTTATACCAGCAA  TTGCAGTTGCCAATACCTATGC  CCAGTCACAGTAGTCGTCACA  TATGCCTCCACCAAGGAGAG  ATTCGCTGGCTGATGCAATCT  GGCACCAAGAACACTGTCTG  TCGTACCCATGACCATAGACTT  ATGGTCAACGACCCTCCAGTA  GCTGACTGTCGGCATATAGGAA  AAGTCCCTTAGCCTTCTCTACC  CCTTCCTCACGCTTCGATCC  AGCCTCATACCCTAATTCGGAT  CCTTTCCGTGGACTTGCTGA  TCTACAAGGGCATTAACCAGGC  AACGTGAAAAGGTGAACACTGA  CATGTCAACCCTCGGACTATCT  CTGCCCCTACTACCAGTCG  CATTCTGACTTTCATCTGGGTGT  GCTCAGCAAACCAAACAAGGTG  TATTGCGGCCAATGTCTTTGC  CACTGGAGTGATAGACTCAACCA  ATTGCTGGCTCATCGCTGT  GGGAAAGTAGTCTCGAAAGTAGC  ACCAAAGTGCAATCAAAGTGGA  ATGAGGGAGTTGGAAGGCTCT |

## Table S3: Standard curves of the two housekeeping genes

| Gene | Ct range | R^2^ | equation |
| --- | --- | --- | --- |
| ACTB | 13.33-28.23 | 0.9853 | y=3.9375x+13.269 |
| GAPDH | 14.10-29.10 | 0.9883 | y=3.9463x+13.976 |

## Table S4: Stability of the two housekeeping genes

| Gene | ACTB | GAPDH |
| --- | --- | --- |
| M | 0.105 | 0.105 |
